# Sunitinib-Induced Hand-Foot Skin Reaction: A Case Report

**DOI:** 10.7759/cureus.114501

**Published:** 2026-08-13

**Authors:** Archana Patel, Rujuta Sanghvi

**Affiliations:** 1 Pharmacology, Sumandeep Vidyapeeth University, Vadodara, IND

**Keywords:** adverse drug reaction (adr), hand-foot syndrome, metastatic rcc, sunitinib, tyrosine kinase inhibitors (tki)

## Abstract

Hand-foot skin reaction (HFSR) is a common dermatological adverse effect of multikinase inhibitors like sunitinib that can impair quality of life and disrupt treatment continuity. We report a 52-year-old male with metastatic renal cell carcinoma who developed Common Terminology Criteria for Adverse Events (CTCAE v5.0) Grade 2 sunitinib-induced HFSR presenting as painful palmar-plantar hyperpigmented lesions and bullae during his third treatment cycle (50 mg/day; two weeks on, one week off). Sunitinib was temporarily held, and multimodal supportive management (including topical corticosteroids, analgesics, and skin barrier care) was initiated. Following complete resolution of lesions, sunitinib was safely resumed at a reduced dose of 37.5 mg daily with no recurrence of HFSR, maintaining oncological therapy. This case suggests that temporary drug interruption combined with targeted supportive care and step-down dose rechallenge may serve as a manageable strategy for Grade 2 HFSR in select patients. However, the efficacy and safety of this approach cannot be generalized, and further studies are needed to establish standardized rechallenge protocols.

## Introduction

Adverse drug reactions (ADRs) are defined by the World Health Organization as “a response to a medication that is noxious and unintended and occurs at doses normally used in man”. ADRs represent a significant cause of morbidity and healthcare burden, adversely affecting patient safety and quality of life. Among these, cutaneous adverse drug reactions (CADRs) are the most frequently encountered in clinical practice [1].

Hand-foot skin reaction (HFSR) is a distinct CADR primarily caused by oral multikinase inhibitors (TKIs) such as sunitinib and sorafenib [2].Approximately 30% of patients receiving TKI therapy experience HFSR [3].Sunitinib is a multi-targeted TKI that inhibits vascular endothelial growth factor receptors (VEGFR-1, VEGFR-2, VEGFR-3), platelet-derived growth factor receptors (PDGFR-α, PDGFR-β), stem cell factor receptor (KIT), Fms-like tyrosine kinase-3 (FLT-3), colony-stimulating factor-1 receptor (CSF-1R), and the rearranged during transfection (RET) receptor [3,4]. It was approved by the US Food and Drug Administration (FDA) in January 2006 for the treatment of advanced/metastatic renal cell carcinoma (mRCC) and gastrointestinal stromal tumors (GIST) [4,5].

CADRs represent a substantial proportion of TKI-associated toxicities [6]. Unlike classic chemotherapeutic agents (e.g., 5-fluorouracil, capecitabine) that typically induce diffuse palmar-plantar erythrodysesthesia (PPE), sunitinib characteristically produces HFSR, which presents as localized erythematous lesions, hyperkeratotic plaques, and bullae on areas exposed to friction or pressure [2,3]. Recognizing these features and initiating prompt management is crucial to prevent dose reductions or premature discontinuation of vital oncological therapy [7]. Although sunitinib-induced HFSR is well-described, real-world evidence guiding successful re-exposure strategies remains sparse. This case report contributes to the existing literature by documenting the complete clinical course of sunitinib-induced Grade 2 HFSR, detailing the successful resolution following targeted drug dechallenge, safe rechallenge at a reduced dose, and sustained absence of recurrence without sacrificing therapeutic efficacy.

## Case presentation

A 52-year-old male farmer from a rural region of Gujarat (Dahod district), Western India, presented to the outpatient clinic complaining of painful skin lesions with a severe burning sensation on both hands and feet that had been persisting for two days. The symptoms markedly impaired his ability to walk and perform routine daily activities. His medical history was notable for left renal cell carcinoma (RCC) diagnosed seven months prior, which had progressed to stage IV (T3a N0 M1) with pulmonary metastases three months before presentation. He was receiving oral sunitinib 50 mg daily on an optimised modified schedule of two weeks on treatment followed by one week off. He had completed 1.5 months (two cycles) of therapy. The patient maintained an Eastern Cooperative Oncology Group (ECOG) performance status of 1 [8].He had no history of prior chemotherapy, radiation therapy, pre-existing dermatological conditions, peripheral vascular disease, or diabetes mellitus. His concurrent medications included metoclopramide, pantoprazole/domperidone, and multivitamin supplements.

The patient had tolerated the first two treatment cycles without adverse events. However, on day 10 of the third cycle (10 days after starting the third cycle, approximately 45 days after initiating sunitinib, he noted new-onset lesions on his palms and soles. Initially painless, the lesions rapidly became hyperpigmented, accompanied by intense localized dysesthesia and tenderness. He had no personal or family history of similar skin conditions.

On physical examination, the patient was conscious, cooperative, and oriented. His vital signs were within normal limits. Dermatological examination revealed diffuse, blanchable lesions over the palmar aspect of the fingertips bilaterally (Figure 1). Multiple skin-colored to hyperpigmented tender plaques with localized flaccid bullae and underlying erythematous margins were present along the medial border of the right foot (Figure 2), the dorsum of both feet (Figure 3), and the posterior heel/ankle regions (Figure 4). There was no mucosal, nail, or truncal involvement.

**Figure 1:**
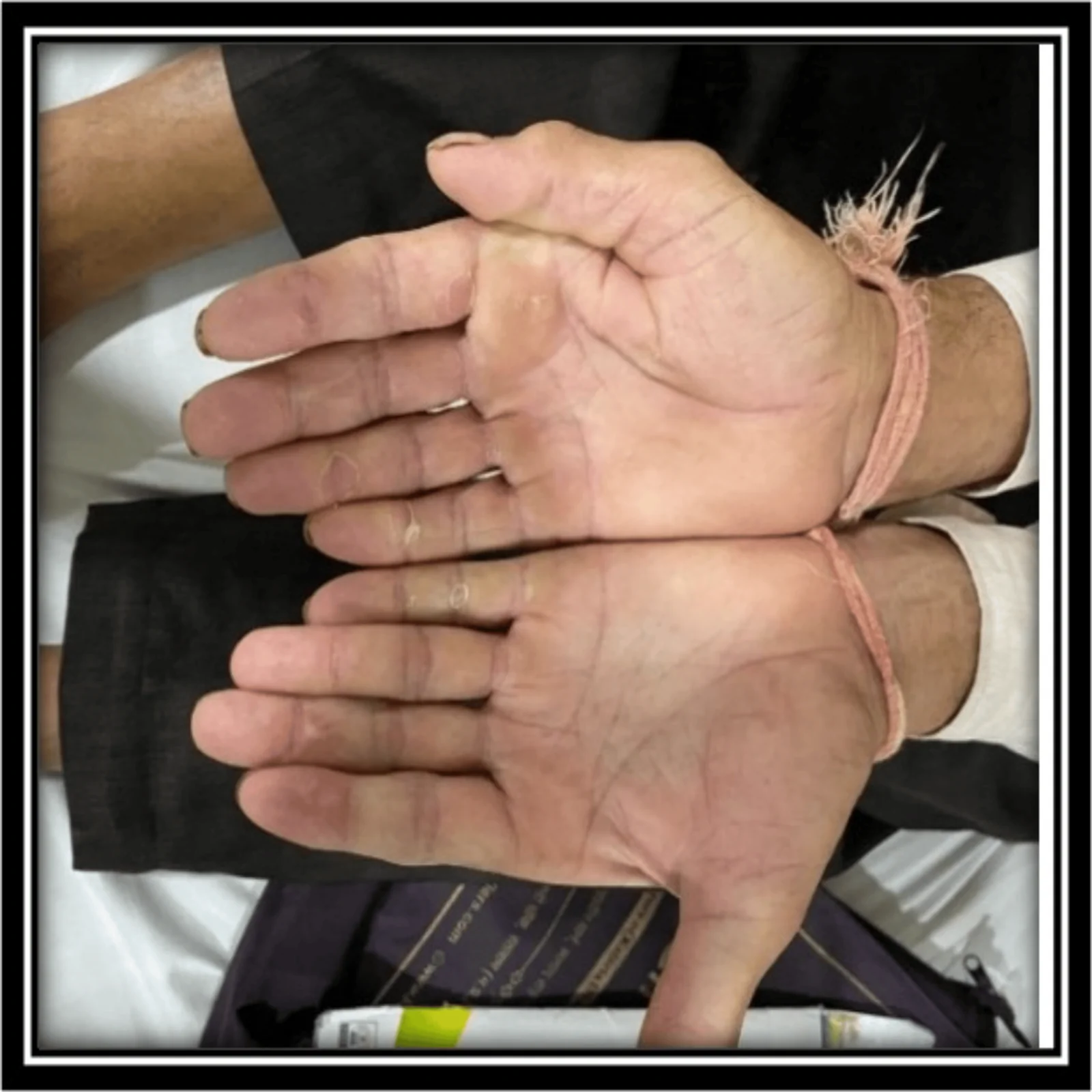
Cutaneous manifestation of Grade 2 sunitinib-induced hand-foot skin reaction (HFSR). Well-demarcated, diffuse blanchable skin-colored to mild erythematous hyperkeratotic plaques on the distal pulp of the digits bilaterally, prominent over pressure-bearing friction sites.

**Figure 2:**
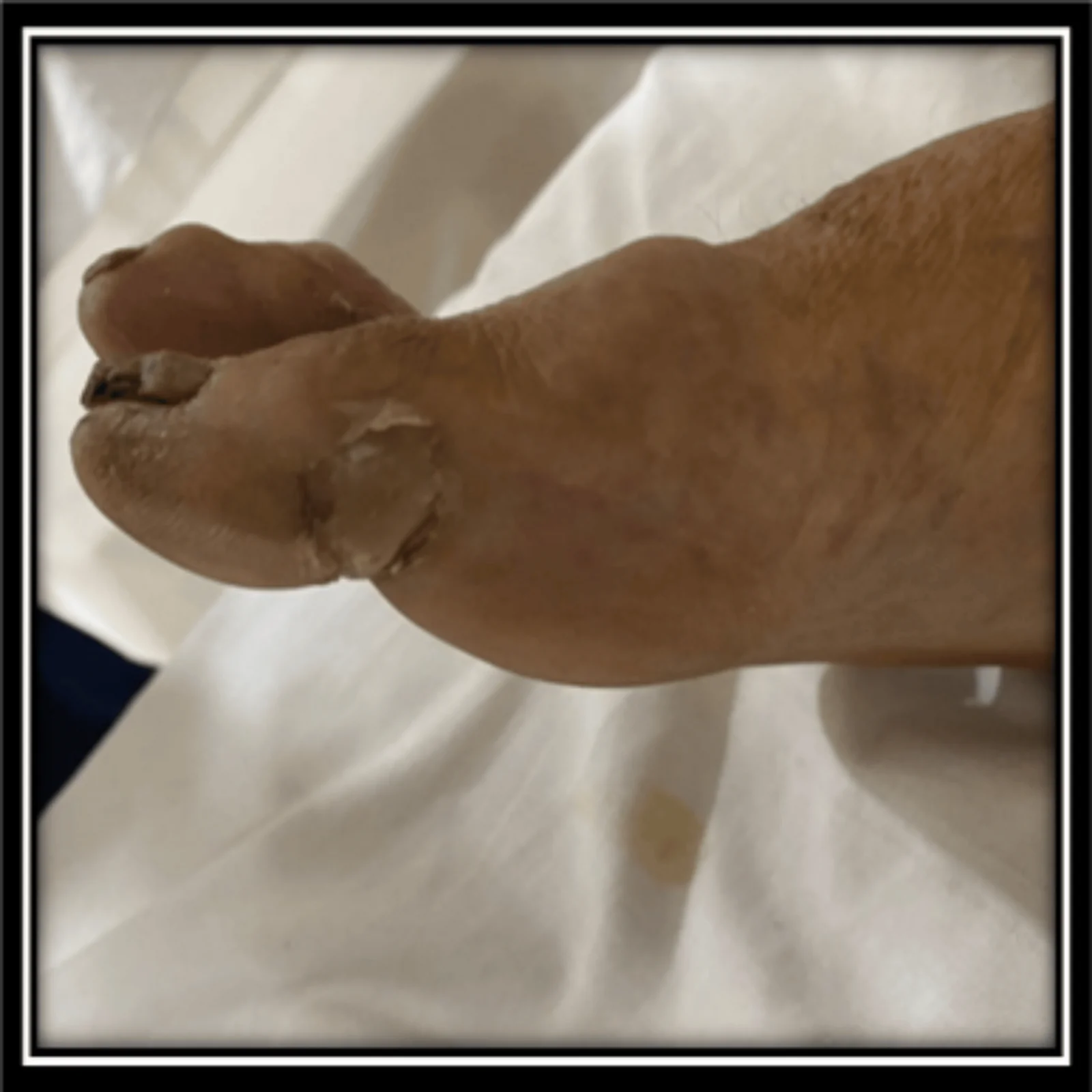
Medial foot involvement in sunitinib-induced hand-foot skin reaction (HFSR). Well-demarcated, skin-colored hyperkeratotic plaque with faint surrounding erythema on the medial aspect of the right foot, located over a prominent mechanical friction bearing site.

**Figure 3:**
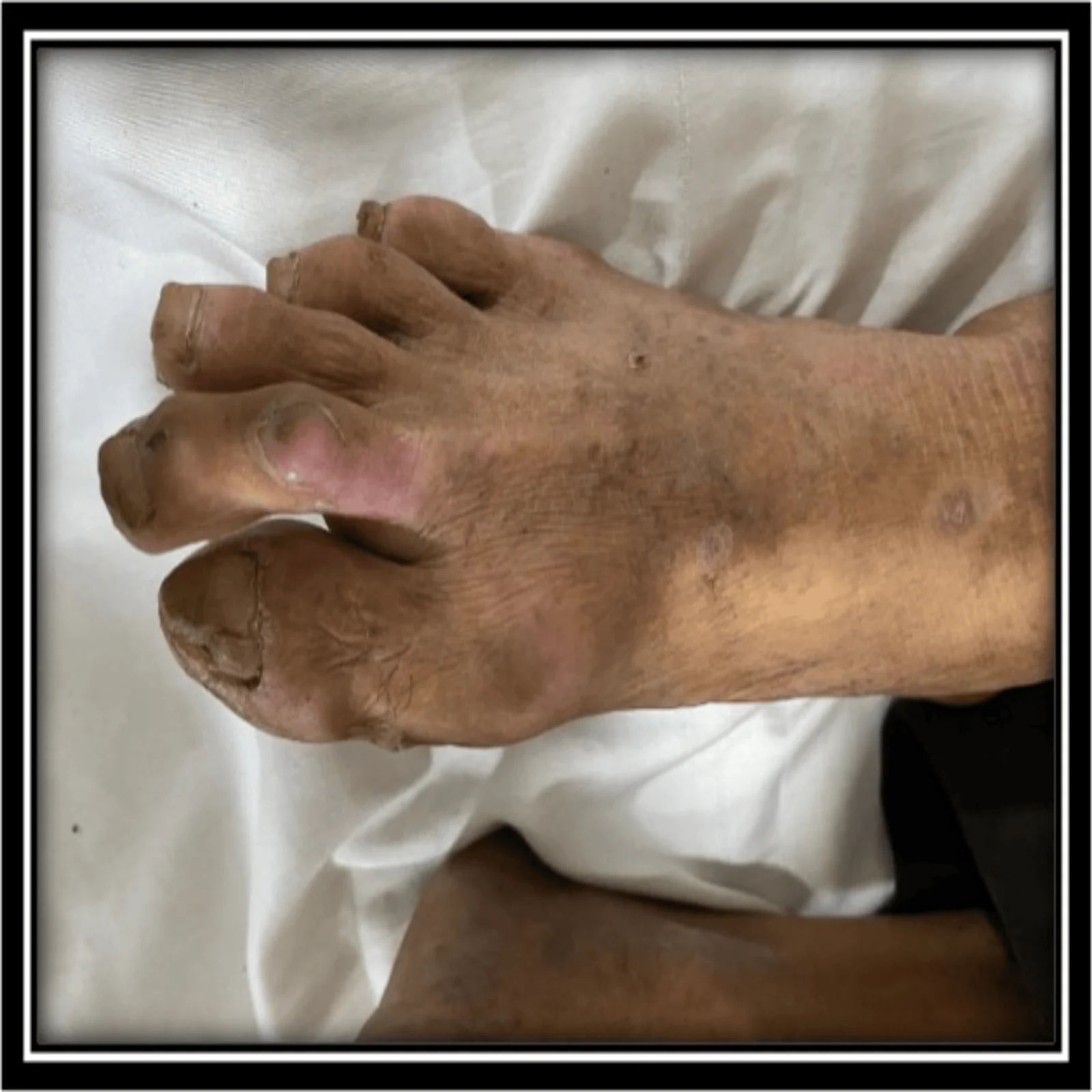
Dorsal foot involvement Well-defined, skin-colored to mild erythematous hyperkeratotic plaque over the dorsum of the foot, exhibiting localized tenderness without epidermal sloughing or ulceration.

**Figure 4:**
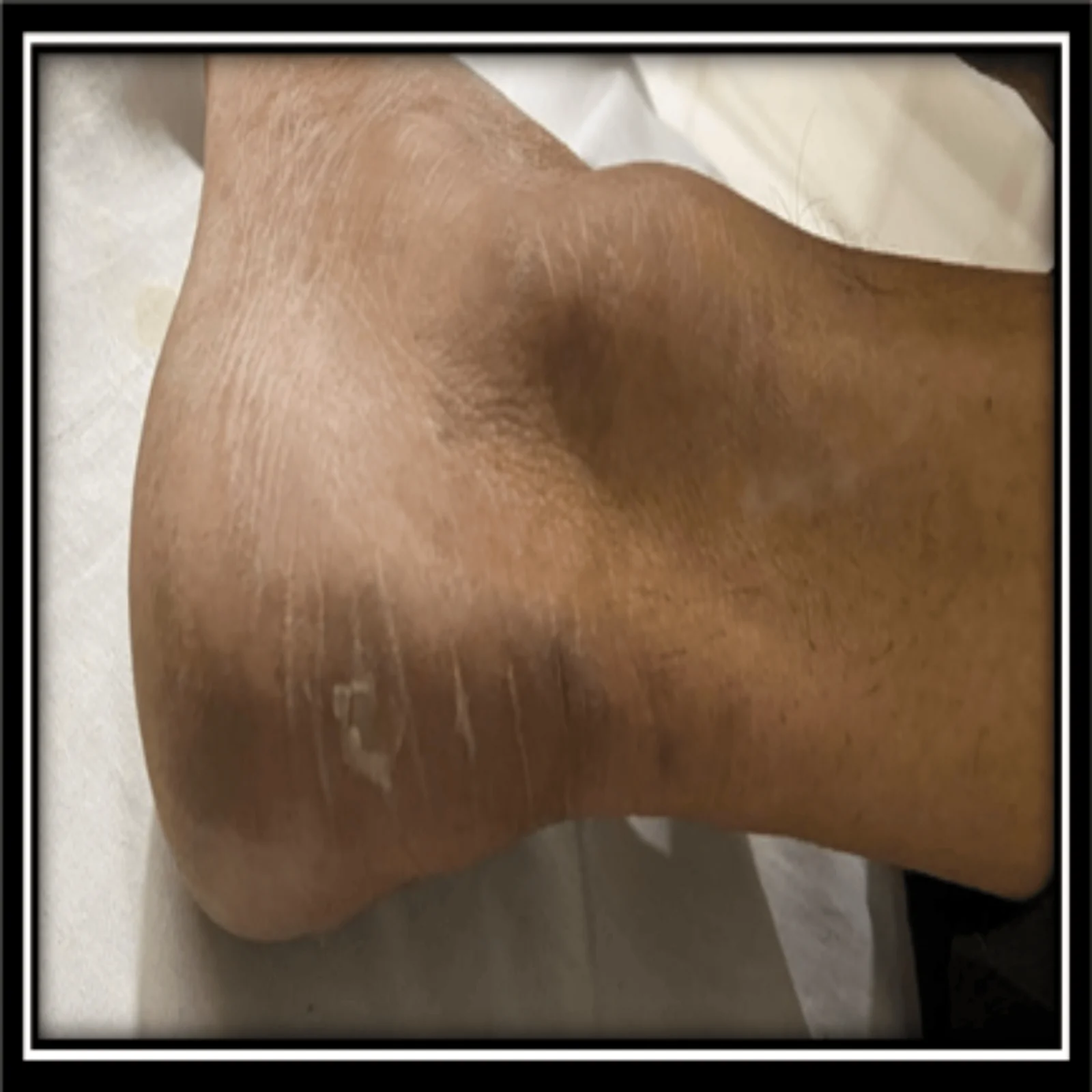
Posterior ankle involvement Thickened, skin-colored hyperkeratotic plaque on the posterior aspect of the ankle joint (Achilles region), a site subject to repetitive friction from footwear.

All baseline laboratory investigations were within normal or clinically expected limits, as summarized in Table 1. A skin biopsy was deemed unnecessary due to the pathognomonic clinical presentation and clear temporal relationship.

**Table 1:** Initial Laboratory Findings with Reference Ranges and Interpretations. AST: Aspartate Aminotransferase; ALT: Alanine Aminotransferase; mRCC: metastatic renal cell carcinoma

Laboratory Parameter	Patient's Result	Reference Range	Interpretation
Hemoglobin	11.2 g/dL	13.0 - 17.0 g/dL	Mild normocytic anemia (common in mRCC)
Total Leukocyte Count	6,800 /μL	4,000 - 11,000 /μL	Normal
Platelet Count	210,000 /μL	150,000 - 450,000 /μL	Normal
Blood Urea	18 mg/dL	10 - 40 mg/dL	Normal
Serum Creatinine	0.6 mg/dL	0.6 - 1.2 mg/dL	Normal renal function
Serum Sodium	138 mEq/L	135 - 145 mEq/L	Normal
Serum Potassium	4.2 mEq/L	3.5 - 5.0 mEq/L	Normal
Serum Bilirubin (Total)	0.8 mg/dL	0.2 - 1.2 mg/dL	Normal
AST / ALT	24 / 28 U/L	10 - 40 U/L	Normal liver enzymes

Differential diagnosis

To establish the diagnosis of sunitinib-induced HFSR, alternative dermatological conditions were systematically excluded based on distinct clinical features [3]. Stevens-Johnson syndrome (SJS) was ruled out due to the strict confinement of lesions to high-friction areas, the absence of mucosal involvement, and the lack of systemic toxicity. Allergic contact dermatitis was similarly excluded based on the clinical history and the absence of topical allergen exposure. Erythromelalgia was disregarded because, while it presents with burning pain and erythema, it is typically triggered by heat, relieved by cold water immersion, lacks hyperkeratotic plaque formation, and exhibits distinct vascular kinetics [9]. Graft-versus-host disease (GVHD) was not considered as the patient had no history of stem cell or solid organ transplantation. Furthermore, erythema multiforme (EM) was excluded due to the absence of classic targetoid lesions and systemic features [3].Based on the Common Terminology Criteria for Adverse Events (CTCAE v5.0), the overall severity of the reaction was graded as Grade 2, defined by painful skin changes and hyperkeratosis interfering with activities of daily living [10].

Management

Sunitinib therapy was temporarily held upon diagnosis, and a multi-mechanistic supportive care regimen was initiated in accordance with established clinical practice guidelines for TKI-induced Grade 2/3 cutaneous toxicity [11]. To rapidly suppress acute tissue inflammation, systemic corticosteroids were prescribed using oral prednisolone on a tapering schedule consisting of 20 mg daily for five days, followed by 15 mg daily for five days, and concluding with 10 mg daily for five days [7,11].Neuropathic analgesia was provided with oral gabapentin 100 mg twice daily for 30 days to target TKI-induced neuropathic pain and burning dysesthesia resulting from peripheral nerve fiber irritation and epidermal microvascular ischemia [12]. Localized pruritus and discomfort were managed with antihistamine therapy using oral levocetirizine 5 mg twice daily for 30 days [11]. While antimicrobial stewardship guidelines generally advocate against prophylactic systemic antibiotics for uninfected intact bullae [13], cefadroxil (500 mg twice daily for five days) was administered selectively in this context as a short-course secondary infection prophylaxis. This decision was guided by the patient’s high-risk occupational profile as a farmer in a rural setting with ongoing exposure to soil and organic pathogens, alongside the presence of tense, flaccid bullae on high-friction, weight-bearing plantaro-palmar surfaces prone to rupture during daily movement. Finally, topical therapy comprised high-potency topical clobetasol propionate 0.05% cream applied twice daily to active plaques, which was supplemented by an emollient cream containing aloe vera and vitamin E to restore epidermal barrier function.

Outcome and follow-up

The patient demonstrated rapid clinical improvement following treatment initiation. The severe burning sensation and neuropathic dysesthesia receded within seven days. Complete re-epithelialization of bullae and resolution of erythematous plaques occurred within 14 days, allowing the patient to resume normal activities of daily living without restriction.

Following complete skin restoration, sunitinib was restarted at a reduced dose of 37.5 mg daily on the same schedule (two weeks on, one week off) for cycle four, following standard clinical guidelines recommending a one-level dose reduction (from 50 mg to 37.5 mg) after resolution of Grade 2 HFSR to prevent recurrent severe toxicity while maintaining therapeutic oncological exposure [14].

The patient tolerated the rechallenge (with reduced dose) well, with no recurrence of cutaneous toxicity. Regular monitoring at the initiation and midpoint of subsequent cycles allowed uninterrupted oncological treatment. 

Applying the Naranjo Adverse Drug Reaction Probability Scale, the case yielded a score of 8 (indicating a "probable" reaction), supported by the temporal sequence, symptom resolution upon drug withdrawal, and absence of recurrence at a lower dose [15]. The World Health Organization-Uppsala Monitoring Centre (WHO-UMC) causality assessment system similarly classified the event as "probable" [16].

## Discussion

Multikinase inhibitors such as sunitinib and sorafenib are well-documented triggers of HFSR [2].Clinical onset typically begins with dysesthesia, tingling, or numbness, progressing to burning pain, well-demarcated erythematous lesions, localized edema, hyperkeratosis, and bullae formation on pressure points [3].These skin changes cause significant physical discomfort and functional impairment [7].

The exact pathophysiology of HFSR remains incompletely understood and is likely multifactorial [2,3,6]. One hypothesis suggests that drug accumulation occurs in areas with high density of eccrine sweat glands, such as the palms and soles [2,3]. Another widely accepted theory posits that mechanical microtrauma breaks down sub capillary structures, allowing TKI leakage into localized soft tissue, triggering a potent inflammatory response [3,6].

Primary management of HFSR begins prior to and during treatment. Recommended measures include pre-treatment podiatric evaluation to remove hyperkeratotic skin/calluses, avoidance of mechanical friction and heat, wearing thick cotton socks and supportive footwear, and daily application of keratolytic emollients [3,7].

For CTCAE Grade 2 HFSR (painful skin changes interfering with instrumental activities of daily living), standard guidelines recommend initiate conservative measures: high-potency topical corticosteroids, topical analgesics, and intensive moisturization [3,7,10]. According to package inserts and clinical practice guidelines, persistent or intolerable Grade 2 HFSR warrants temporary withholding of sunitinib until toxicity resolves to Grade 0-1, followed by dose reduction (e.g., from 50 mg/day down to 37.5 mg/day, and subsequently 25 mg/day if needed) [3,10].

Our case offers valuable insights when compared to existing literature. For instance, Das et al. (2020) [3] described a similar case of sunitinib-induced HFSR in a patient with RCC; however, causality could only be established as "probable" with a Naranjo score of 5 because formal rechallenge details were omitted. In contrast, our report documents a controlled dechallenge and successful rechallenge at a reduced dose (37.5 mg/day), yielding a Naranjo score of 8 [15] and WHO-UMC "probable" categorization [16].

To address the severe inflammatory and neuropathic components in this patient, an individualized multi-mechanistic regimen was implemented alongside standard care. This included short-course systemic steroids to rapidly control acute inflammation, gabapentin specifically targeting severe dysesthesia, high-potency topical corticosteroids, and prophylactic oral antibiotics for intact friction bullae.

While individual case observations cannot establish treatment efficacy or direct causality, this multimodal regimen was associated with favorable clinical improvement without secondary infectious complications, supporting a timely resumption of sunitinib at the reduced 37.5 mg/day dose.

## Conclusions

This case underscores the vital role of a multidisciplinary team - comprising clinical pharmacologists, dermatologists, and oncologists - in the early recognition and proactive management of sunitinib-induced severe HFSR. Although Grade 2 HFSR can significantly impair patient mobility and quality of life, it should not automatically necessitate the permanent cessation of life-extending targeted kinase inhibitor therapy. Following a temporary two-week drug holiday combined with targeted supportive measures-including short-course systemic steroids, gabapentin for dysesthesia, high-potency topical corticosteroids, and prophylactic antibiotics-sunitinib was safely resumed at a reduced dose of 37.5 mg/day. This tailored regimen achieved complete cutaneous restoration without disease progression, demonstrating that strategic dose modification and supportive care can maintain essential oncologic treatment while preserving patient safety and quality of life.
